# *Masculinizer* and *Doublesex* as Key Factors Regulate Sexual Dimorphism in *Ostrinia furnacalis*

**DOI:** 10.3390/cells11142161

**Published:** 2022-07-11

**Authors:** Honglun Bi, Xiaowei Li, Xia Xu, Yaohui Wang, Shutang Zhou, Yongping Huang

**Affiliations:** 1 State Key Laboratory of Cotton Biology, School of Life Sciences, College of Agriculture, Henan University, Kaifeng 475004, China; honglunbi@henu.edu.cn; 2 Key Laboratory of Insect Developmental and Evolutionary Biology, Center for Excellence in Molecular Plant Sciences, Shanghai Institute of Plant Physiology and Ecology, Chinese Academy of Sciences, Shanghai 200032, China; cqdxlxzz2020@cqu.edu.cn (X.L.); xuxia@zaas.ac.cn (X.X.); yhwang@cemps.ac.cn (Y.W.)

**Keywords:** CRISPR/Cas9, *Masculinizer*, *doublesex*, sexual dimorphism, *Ostrinia furnacalis*

## Abstract

**Simple Summary:**

In animals, sexually dimorphic traits are ubiquitous and play vital roles in reproduction, courtship, and environmental adaptation, especially in insects. In this study, we used the CRISPR/Cas9 genome editing system to generate somatic mutations of the *Masculinizer* (*Masc*) and *doublesex* (*dsx*) genes in the sex determination pathway of *Ostrinia furnacalis*. The *OfMasc* and *Ofdsx* genes are structural orthologs of the key sex regulation factors in *Bombyx mori*. Mutation of the *OfMasc* and *Ofdsx* genes induced abnormal external genitalia, adult sterility, and sex reversal of sexually dimorphic traits including wing pigmentation, gene expression patterns, and *dsx* sex-specific splicing. These results demonstrate that the *Masc* and *dsx* genes are conserved factors in sexually dimorphic traits, and therefore represent potential target genes in the effort to control *O. furnacalis* and other lepidopteran pests.

**Abstract:**

Sex determination is an important and traditional biological process. In Lepidoptera, *Masculinizer* (*Masc*) and *doublesex* (*dsx*) are the essential genes for sex determination and play critical roles in sexual differentiation and development. The functions of *Masc* and *dsx* have been characterized in several model insect species. However, the molecular mechanism and sex determination functions of *Masc* and *dsx* in *Ostrinia furnacalis*, an agricultural pest, are still unknown. Here, we successfully used the CRISPR/Cas9 genome editing system to knock out *OfMasc* and *Ofdsx*. Mutation of *OfMasc* induced male external genital defects and sterility. Disruptions of the *Ofdsx* common region caused sex-specific defects in the external genitals and adult sterility. In addition, we found that *OfMasc* and *Ofdsx* can regulate the pigmentation genes that control wing pigmentation patterns. These results demonstrate that *OfMasc* and *Ofdsx* play key roles in the sex determination of *O. furnacalis*, and suggest novel genetic control approaches for the management of pests, including *O. furnacalis*.

## 1. Introduction

Sexually dimorphic traits are ubiquitous in plants and animals. Most animal species consist of two distinct sexes, and the differences between male and female animals are numerous and pronounced at the morphological, physiological, and behavioral levels [1]. However, sexual dimorphism presents a question: how can a genome largely shared between the sexes give rise to such different forms [2]? A compelling answer to this question is provided by the sex-specific expression of shared genes [3]. Sex determination is an essential and hierarchically regulated biological process with high diversity in different organisms, including insects [4,5,6]. Sexually dimorphic traits, including body size, pigmentation, external genitals, sex-specific behavior and physiology are prevalent across the animal kingdom and especially in insect species [7]. Sexual dimorphism typically manifests in differences in body and wing color [1].

In animals, the *doublesex* (*dsx*) gene, the *mab-3* gene and the Dsx- and mab-3-related transcription factor 1 (*Dmrt1)* are three homological factors that, through female and male specific expression and splicing, play an important role in the regulation of sexually dimorphic traits in *Drosophila melanogaster*, *Caenorhabditis elegans*, and mammalian species [8,9,10]. In insect species, sex determination plays a key part in biological development and reproduction [5,11]. In *D. melanogaster*, the *Sex lethal* (*Sxl*) gene is initially regulated by the X:A ratio [12]. Then, the *dsx* gene, which is a conserved downstream gene involved in sex determination, regulates sexual differentiation [13,14].

In *Drosophila*, the HOX protein Abdominal-B (ABD-B) and the sex-specific isoforms of DSX directly regulate the *bab* cis-regulatory element (CRE) to induce sexually differentiated pigmentation on abdominal segments [15]. In dragonflies, there is sexually dimorphic coloration; for instance, in *Ischnura senegalensis*, females are orange and males are blue. However, the molecular mechanism that regulates this difference is unclear [16]. In locusts, such as the gregarious *Schistocerca gregaria*, the body color of males and females does not differ in the nymphal stage; when male locusts reach sexual maturity, however, their male abdomen and legs turn yellow [17]. However, despite the fact that sexually dimorphic coloration is widespread in insects, the molecular mechanisms that regulate it are not well understood, and it is also unclear how the sex determination pathway regulates pigmentation.

In Lepidoptera, studies of sex determination have mainly focused on the silkworm *Bombyx mori*, which is an important lepidopteran model insect [18,19,20]. A previous study showed that the *Masculinizer* (*Masc*) gene is repressed by *Fem* piRNA in female silkworm [21]. Moreover, the *Masc* gene controls *Bmdsx* gene splicing in *B. mori* [22,23]. Mutation in *Masc* induces the appearance of female characteristics, including female-specific ventral chitin plates and genital papillae in male individuals [24]. Furthermore, disruption of the *Bmdsx* gene induces abnormal gonads and external genitalia, and sex-specific sterility [24,25]. In the Asiatic corn Borer, *Ostrinia furnacalis* (Lepidoptera: Pyralidae), which is one of the most destructive pests of corn, especially in China and northeast Asia [26,27], the *Masc* gene and the *dsx* gene have been described in previous studies [28,29,30,31]. The *OfMasc* gene is regulated by the endosymbiotic bacterium *Wolbachia*; a failure dosage compensation induces male lethality [28,30]. However, the genetic and functional relationships between these genes in *O. furnacalis* sex determination and differentiation are still unclear.

In our study, we used the CRISPR/Cas9 genome editing system to generate somatic mutations in the *Masc* and *dsx* genes in the sex determination pathway of *O. furnacalis*. The *OfMasc* and *Ofdsx* genes are structural orthologs of the key sex regulation factors in *B. mori*. Mutation of the *Ofdsx* gene induced abnormal external genitalia, adult sterility, and sex reversal of sexually dimorphic traits, including wing pigmentation, gene expression patterns, and *dsx* sex-specific splicing. These results demonstrate that the *Masc* and *dsx* genes are the conserved factors in sexually dimorphic traits, and therefore represent potential target genes for research into the control of *O. furnacalis* and other lepidopteran pests.

## 2. Materials and Methods

### 2.1. Insect Strains and Rearing

A laboratory strain of *O. furnacalis* was reared on an artificial diet (Table 1) under standard conditions in an incubator, at a temperature of 25 °C and with a 16:8 h light:dark cycle [32]. *O. furnacalis* pupae were sexed, and the emerging adults were mixed in transparent air-filled plastic bags to mate with each other and lay eggs [26]. 

Preparation method: Boil 900 mL of water mixed with agar, add other ingredients, mix well, and store in the refrigerator after cooling.

### 2.2. Phylogenetic Analysis

Phylogenetic relationships were determined based on sequence alignment (DNAMAN 8.0 software) and phylogenetic analysis using Mega 5 [33,34]. All ambiguous positions were removed for each sequence pair. The neighbor-joining method was used to create a tree from 9 available MASC protein sequences, and the reliability of the tree was tested by bootstrap analysis with 1000 replications. The GenBank accession numbers and references of the protein sequences are as follows: *B. mori* (BAO79517.1), *Trilocha varians* (BAS02075.1), *Helicoverpa armigera* (QCD63870.1), *Agrotis ipsilon* [35], *Plutella xylostella* [36], *O. furnacalis* (BAS02074.1), *Ephestia kuehniella* (QXE45293.1), *Artemia franciscana* (ARB66312.1), *Artemia parthenogenetica* (ARB66313.1).

### 2.3. Quantitative Real-Time PCR (qRT-PCR)

For qRT-PCR analyses, total RNA was extracted from *O. furnacalis* larvae and adults using Trizol reagent (Invitrogen, Carlsbad, CA, USA) and treated with RNase-free DNase I (Ambion, Austin, TX, USA), according to the manufacturer’s instructions. cDNAs were synthesized using the Omniscript Reverse Transcriptase kit (Qiagen, Hilden, Germany) in a 20 μL reaction mixture containing 1 μg total RNA. qRT-PCR analysis for *OfMasc* and *Ofdsx* mutants was performed using a SYBR Green Real-Time PCR Master Mix (Thermo Fisher Scientific, Waltham, MA, USA) on an Eppendorf Real-Time PCR System. The PCR conditions were as follows: initial incubation at 95 °C for 5 min, 40 cycles at 95 °C for 15 s and 60 °C for 1 min. *O. furnacalis actin* was used as an internal control [32]. The gene-specific primers used for qRT-PCR are listed in Table 2.

### 2.4. In Vitro Transcription of Cas9 mRNA and sgRNA

We selected two 23-bp sgRNAs targeting *OfMasc* and one sgRNA targeting *Ofdsx*. The sgRNAs were sub-cloned into the 500-bp linearized CloneJet PJET1.2-T vector (Thermo Fisher Scientific) upstream of the protospacer adjacent motif (PAM) sequence, to allow sgRNA expression under the control of the T7 promoter. The sgRNAs were synthesized in vitro using a MEGAScript T7 kit (Ambion), according to the manufacturer’s instructions. Cas9 mRNA was synthesized in vitro using the mMESSAGE T7 Kit (Ambion) and a PTD1-T7-Cas9 vector as the template [35], according to the manufacturer’s instructions.

### 2.5. Microinjection of Embryos

Mated female *O. furnacalis* moths were allowed to lay eggs on transparent plastic bags. A previously reported microinjection method was employed [37]. Within 1 h of oviposition, the eggs were injected on the lateral side with a mixture containing 300 ng/μL of Cas9 mRNA and 150 ng/μL sgRNA. After injection, the eggs were incubated in a humidified chamber at 25 °C for 4 days until hatching.

### 2.6. Genomic DNA Extraction and Identification of Mutagenesis

The genomic DNA was extracted from the newly hatched larvae, incubated with proteinase K, and purified via a standard phenol:chloroform extraction and isopropanol precipitation extraction, followed by RNase A treatment. A PCR was carried out to identify *OfMasc* and *Ofdsx* mutant alleles using primers F1 and R1 (Table 2) spanning the target site in *OfMasc* and *Ofdsx*. The PCR conditions were as follows: 98 °C for 2 min, followed by 35 cycles of 94 °C for 10 s, 55 °C for 30 s, and 72 °C for 1 min, followed by a final extension period of 72 °C for 10 min. The PCR products were sub-cloned into the CloneJet PJET1.2-T vectors (Thermo Fisher Scientific) and sequenced. The PCR products were also used for the T7 endonuclease I (T7EI) assay as previously described [38]. The mutants were photographed with a digital stereoscope (Nikon AZ100, Tokyo, Japan).

### 2.7. Hatchability Assay

In order to evaluate the hatchability of *Masc* and *dsx* mutants, the males and females with *OfMasc* and *Ofdsx* mutations were crossed with mutant moths and virgin wild type male and female moths. Five pairs of moths were collected for one group. Hatchability assays of each group were repeated 3 times. After female moths laid eggs for two days, the eggs of each pair were collected and incubated in a humidified chamber at 25 °C for 4 days until hatching. The hatching rates were analyzed.

### 2.8. Statistical Analysis of Data

The data were analyzed using GraphPad Prism (version 5.01) with one-way analysis of variance, the Dunnett post hoc test and Bonferroni analysis. Error bars stand for the means ± SEM, and three asterisks stand for *p <* 0.001.

## 3. Results

### 3.1. Phylogenetic Analysis of MASC and DSX Proteins in O. furnacalis

The phylogenetic tree was constructed using sequences of OfMASC and MASC protein sequences from six different lepidopteran insects, namely *H. armigera*, *B. mori*, *T. varians*, *A. ipsilon*, *P. xylostella*, and *E. kuehniella*, and two other species, namely *A. franciscana* and *A. parthenogenetica* (Figure S1B). The phylogenetic tree showed that OfMASC was closest to *P. xylostella* and *E. kuehniella* MASC, suggesting a conserved function. Subsequently, the amino acid sequence of the OfMASC protein was compared with the other lepidopteran MASC proteins. The analyzed multiple alignment results show that the OfMASC protein has two tandem CCCH-type zinc finger (ZF) domains, a bipartite nuclear localization signal (bNLS), and a masculinization domain (MD) (Figure S1A). Then, we used the NCBI BLAST program to find the amino acid sequences of DSX proteins in the NCBI database and constructed the phylogenetic tree of DSX (Figure S2). The analyzed results showed that the OfDSX protein was closest to *Galleria mellonella*, a moth of the same superfamily Pyraloidea as *O. furnacalis*, and clustered with the DSX proteins of other lepidopteran moth insects, suggesting a conserved function.

### 3.2. CRISPR/Cas9-Mediated Mutagenesis of OfMasc and Ofdsx

**Figure 1:**
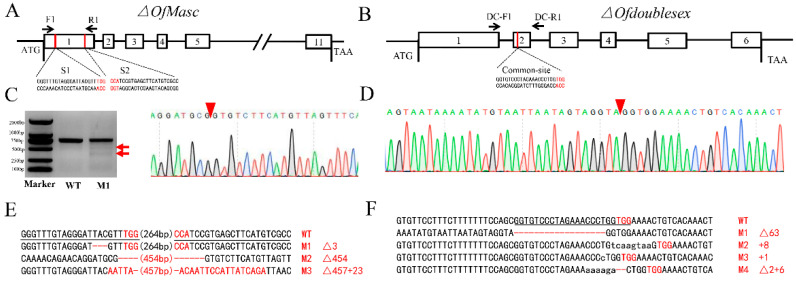
CRISPR/Cas9-mediated mutations in the *OfMasc* and *Ofdsx* target sites. (**A**) The two target sites of the *OfMasc* genome locus focused on the first exon. (**B**) The target site of the *Ofdsx* genome locus focused on the second exon of the common region in the female and male spliced variants. (**C**) T7 endonuclease I treatment of extracts of wild type (WT) and mutant (M) pupae of *OfMasc* and genomic sequencing demonstrate alterations at the target site. Arrows indicate the two bands observed in mutants. The red wedge indicates the position of cleavage by the CRISPR/Cas9 genome editing system. (**D**) Sequencing chromatogram of the *Ofdsx* mutants. The red wedge indicates the position of cleavage by the CRISPR/Cas9 genome editing system. (**E**) *OfMasc* mutations detected by sequencing. The PAM sequence is in red. The black line represents the target sites. (**F**) *Ofdsx* mutations detected by sequencing. The PAM sequence is in red. The black line represents the target site.

In order to investigate the function of these two sex determination genes, the high-efficiency genome editing system CRISPR/Cas9 was used to disrupt the *OfMasc* and *Ofdsx* genes. Following the GGN_19_GG rule for sgRNA design [35], we designed two sgRNAs targeting the *OfMasc* and one sgRNA targeting the *Ofdsx* gene. Two targeted sgRNAs were at the exon 1 of the *OfMasc* gene locus (Figure 1A), and one targeted sgRNA was at the exon 2 of the common region of *Ofdsx* female and male transcript isoforms (Figure 1B). The fresh eggs, which were not more than 1 h older, were collected for microinjection. The Cas9 mRNA mixed with *OfMasc* or *Ofdsx* sgRNAs transcripted by a T7 promoter, was prepared according to previous reports [39]. To identify the mutated alleles of the *OfMasc* and *Ofdsx* genes, genomic DNA was extracted as phenotypic expression involved in mutagenesis was shown. The results of the genome sequences indicated that the successful deletion of sequences had taken place between the two target sites in the *OfMasc* gene and deletion in the *Ofdsx* gene (Figure 1).

### 3.3. Disruption of OfMasc and Ofdsx Genes Induced Abnormal External Genitalia and Pigmentation

**Figure 2:**
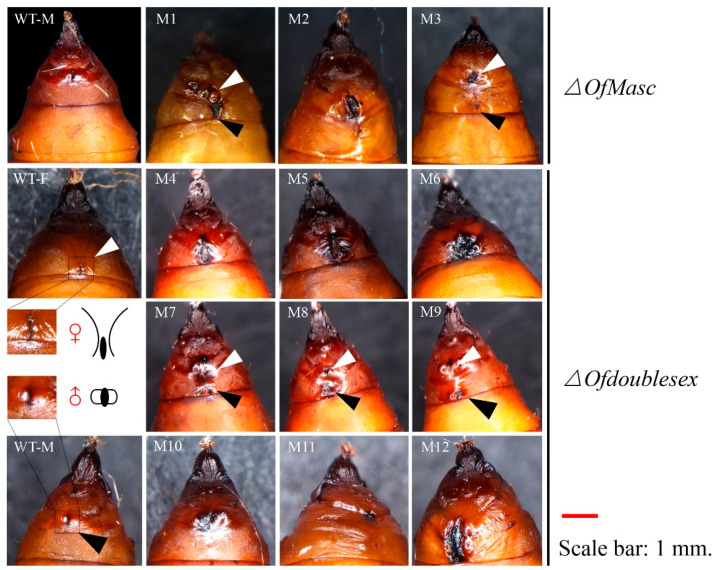
The morphologies of wild type compared with *OfMasc* and *Ofdsx* mutant pupae. In the wild type, the females and males have different gonopore characteristics in pupa morphology; these variations are key to differentiating males from females. Female pupae have an X-shaped line and small crevice in the eighth abdominal segment, whereas male pupae develop two prominent points at the abdomen end in the ninth abdominal segment. The *OfMasc* and *Ofdsx* mutants showed abnormal or defected morphologies. M1–M3 are the male mutants of *Masc* in *O. furnacalis*. In the *dsx* mutants, M4–M6 are the female mutants; M7–M9 are the intersex mutants; M10–M12 are the male mutants. Scale bar: 1 mm.

The mutants displayed some abnormal phenotypes of external genitalia in the pupal stage. In the wild type, females and males have distinct gonopore characteristics in pupa morphology, which are key to distinguishing between females and males. Female pupae have an X-shaped line and a small crevice in the eighth abdominal segment, whereas male pupae develop two prominent points at the abdomen end of the ninth abdominal segment (Figure 2). Because of the key role of sex determination genes in regulating sexual dimorphic traits, the mutant sex determination genes result in abnormal female and male morphological characteristics and sex reversal [40]. For *OfMasc* mutant pupae, we found that there were some abnormal phenotypes, such as deformed gonopores, but only in the male mutants (Figure 2). Moreover, in mutant 2 (M2) and M3, there were some female specific characteristics similar to the X-shaped line. The gender of these male mutants was identified after eclosion. In the *Ofdsx* mutant pupae, we found there were three types of abnormal phenotypes. The *Ofdsx* female mutant had an abnormal X-shaped line, similar to M4, M5, and M6 (Figure 2), and the *Ofdsx* male mutant had defective gonopores, such as in M10, M11 and M12 (Figure 2). Some *Ofdsx* mutant pupae, such as M7, M8 and M9, also had two gonopore characteristics that differed between females and males. We named this mutant type DSX-FM (Table S1).

**Figure 3:**
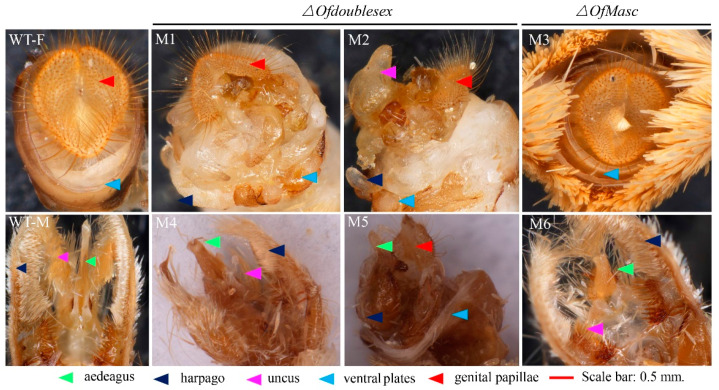
The external genital morphology of the wild type compared with *OfMasc* and *Ofdsx* mutants. In the wild type, adult male external genitalia mainly consist of a harpago, some uncuses and an aedeagus. Female external genitalia mainly consist of a genital papilla and a ventral plate. The male-specific external genitalia in *OfMasc* and *Ofdsx* mutant males exhibited severe structural defects, and the genital papilla and ventral plate were not present and ectopic in *Ofdsx* mutant females. Scale bar: 0.5 mm.

When the mutant pupae entered the adult stage, we found that there were some instances of abnormal external genitals. In the wild type, adult male external genitalia mainly consist of a harpago, some uncuses and an aedeagus. Female external genitalia mainly consist of a genital papilla and a ventral plate. In *OfMasc* mutants, the external genitals were normal in the females and abnormal in the males, which presented with a shorter aedeagus and an abnormal harpago (Figure 3M6). In the female *Ofdsx* mutants, there were some defective genital papillae and aedeagi (Figure 3M1,M2); abnormal harpago and female-specific genital papillae appeared in male *Ofdsx* mutants (Figure 3M4,M5).

**Figure 4:**
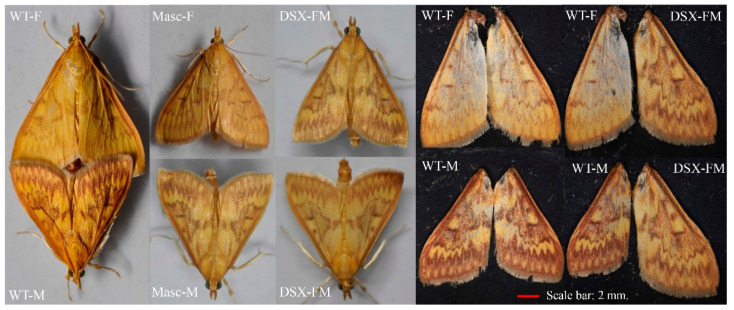
The pigmentation of the wild-type individuals compared with the *OfMasc* and *Ofdsx* mutants. In the wild type, the wing color of males is deeper than that of females. In the *OfMasc* mutant males, the wing color was weaker than in wild-type males. In the *Ofdsx* mutants, the wing color had a level of pigmentation between that of the wild-type males and females. Scale bar: 2 mm.

We also found some other sexual dimorphism trait changes in the adult stage. In the wild type, the wing color of males is deeper than that of females. In the *OfMasc* mutants, however, the wing color of males was weaker than that of wild-type males and similar to that of wild-type females (Figure 4). In the *Ofdsx* mutants, we found the DSX-FM mutants showed more pronounced wing color and stripes than those of wild-type females, but weaker than those of wild-type males (Figure 4). The results suggest that the *dsx* gene regulates pigmentation in *O. furnacalis*.

### 3.4. OfMasc and Ofdsx Mutations Induce Sterility in O. furnacalis 

We then analyzed the fertility data of mutants. We found that deletion of the *OfMasc* gene induced male lethality at the embryonic stage. We collected data concerning sex ratios in the adult stage three times. The percentages of sex ratios of female adults in the total population were about 78%, 85%, and 92% (Figure S4A). In order to analyze the physiological changes in *OfMasc* and *Ofdsx* mutants, we investigated the reproductive ability and hatching rate of the embryos produced when the mutants mated with each other. Because of the defects in the *OfMasc* male mutants’ external genitals, these males could not mate with wild-type females or with *OfMasc* female adults, and no eggs were hatched (Figure S4B). In the *Ofdsx* mutants, both the △DSX-F and △DSX-M individuals all had abnormal external genitals; as such, the *Ofdsx* mutants had no reproductive ability and no next-generation eggs were hatched (Figure S4C).

### 3.5. Detection of Sex-Specific Gene Expression in OfMasc and Ofdsx Mutants

In order to explain these mutant phenotypes, we used the RT-PCR to determine the *Ofdsx* gene expression. In the wild type, the *Ofdsx* female-specific isoform was longer than the male-specific isoform; the specific bands present the female or male *Ofdsx* expression. In the mutants, however, we found that the *OfMasc* male mutants had two bands in one lane, and the *Ofdsx* mutants also had non-single bands in corresponding lanes (Figure S3). These results demonstrate that the mutation of the *OfMasc* gene induces the appearance of female-specific *Ofdsx* isoforms in males.

**Figure 5:**
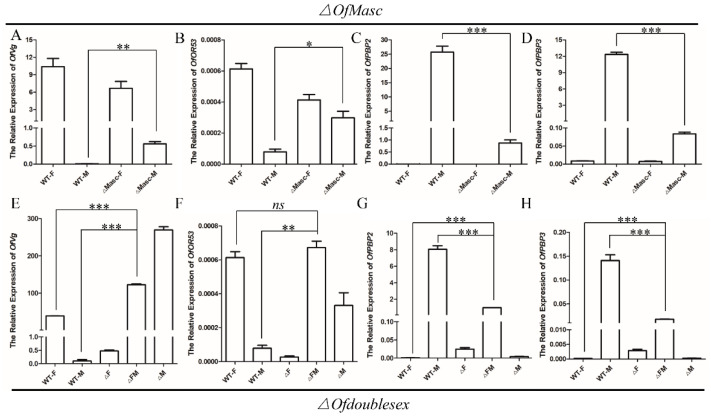
qRT-PCR analysis of the putative downstream genes of *Ofdsx* in the *OfMasc* and *Ofdsx* mutants. (**A**–**D**) Relative mRNA expression levels of *OfVg*, *OfOR53*, *OfPBP2*, and *OfPBP3* in *OfMasc* mutants. (**E**–**H**) Relative mRNA expression levels of *OfVg*, *OfOR53*, *OfPBP2*, and *OfPBP3* in *Ofdsx* mutants. Three individual biological replicates were performed using qRT-PCR. Error bar: SD; *, ** and *** represent significant differences at the 0.05, 0.01 and 0.001 levels (*t*-test) compared with the control.

To investigate whether the disruption of sex-specific *OfMasc* and *Ofdsx* transcripts influences the expression of known sex-biased genes in *O. furnacalis*, we examined the female-biased *Vitellogenin* (*OfVg*) and the *Olfactory Receptor 53* (*OfOR53*) genes, which encode a protein essential for oogenesis and the reception of outside information; we also examined two male biased genes, *Pheromone Binding Protein 2* (*OfPBP2*) and *Pheromone Binding Protein 3* (*OfPBP3*) [32]. Compared with the wild-type males, the relative mRNA expression levels of *OfVg* and *OfOR53* were significantly up-regulated in *OfMasc* and *Ofdsx* male mutants (Figure 5A,B,E,F); in *Ofdsx* mutant females, meanwhile, the levels of *OfVg* and *OfOR53* were significantly decreased (Figure 5E,F). The relative mRNA expression levels of *OfPBP2* and *OfPBP3* were significantly down-regulated in *OfMasc* and *Ofdsx* male mutants (Figure 5C,D,G,H), but were significantly increased in *Ofdsx* female mutants (Figure 5G,H). These results demonstrate that *OfVg*, *OfOR53*, *OfPBP2*, and *OfPBP3* are direct or indirect targets of *dsx* in *O. furnacalis*, which is consistent with previous reports [24,41,42].

**Figure 6:**
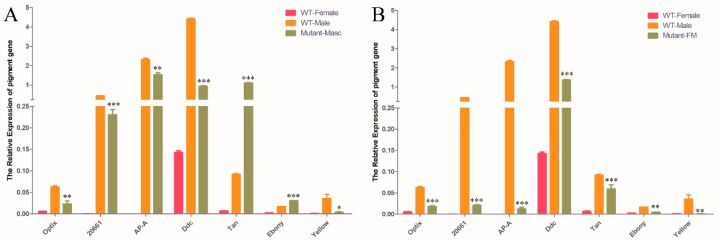
The relative expression of pigmentation genes in *OfMasc* male mutants (**A**) and *Ofdsx* FM mutants (**B**). Three individual biological replicates were performed using qRT-PCR. Error bar: SD; n.s., *, ** and *** represent significant differences at the 0.05, 0.01 and 0.001 levels (*t*-test) compared with the control.

To answer the question of how wing pigmentation is regulated by the mutagenesis of *OfMasc* and *Ofdsx*, we analyzed the relative transcription levels of genes in the melanin synthesis pathway, as well as some other pigmentation-related genes in adult *OfMasc* and *Ofdsx* mutants. In the wild type, the wing pigmentation of males is deeper than that of females, meaning that some pigmentation genes have different expression patterns for females and males. Through the qRT-PCR analysis, we found there were some highly expressed genes in males, including *optix*, *20661*, *apterous* A (*AP-A*), *Ddc*, and *Tan*. High expression caused a deepening of pigmentation in males. In the *OfMasc* male mutants, however, we found that these genes, including *optix*, *20661*, *AP-A* and *Ddc*, were down-regulated compared to wild-type males (Figure 6A). Moreover, in the *Ofdsx* mutants, the expressed pattern was similar to that of *OfMasc* male mutants (Figure 6B). These results demonstrate that disruption of *OfMasc* and *Ofdsx* induces a sex reversal of pigmentation phenotypes, and also that the expression of some genes was up- or down-regulated by the *Ofdsx* gene, either through direct or non-direct effects.

## 4. Discussion

In this study, we focused on the sex determination genes *OfMasc* and *Ofdsx*. The phylogenetic analyses of the *OfMasc* and *Ofdsx* genes showed high homology with other insect species (Figures S1 and S2). Using the CRISPR/Cas9 genome editing system, we successfully knocked out these two genes, which are crucial elements of the sex determination pathway (Figure 1). In *OfMasc* mutants, this induced abnormal external genitals in pupal and adult males (Figure 2 and Figure 3), which led to the sterility of these males and an imbalance in the sex ratio (Figure S4). Disruption of the *Ofdsx* common region induced the malformation of female and male external genitals (Figure 2 and Figure 3), which led to the sterility of male and female adults (Figure S4). Regarding sexual dimorphism, mutated *OfMasc* and *Ofdsx* caused a weakening of pigmentation and down-regulation of the pigmentation genes both for the *OfMasc* males and the *Ofdsx* males, while the *Ofdsx* female mutants showed enhanced pigmentation and up-regulation of pigmentation genes (Figure 4 and Figure 6). RT-PCR results showed the expression of female and male specific *Ofdsx* isoforms in the *OfMasc*-M mutants, and in the *Ofdsx*-F and *Ofdsx*-M mutants (Figure S3). The qRT-PCR results demonstrated disruption of *OfMasc* and *Ofdsx* influenced the expression of sex-biased genes (Figure 5). Our study provides direct evidence that *OfMasc* regulates the expression of the *Ofdsx* gene, and that the *Ofdsx* gene regulates the sexual dimorphism of *O. furnacalis*, including characteristics such as pigmentation, external genitals, sex-biased genes, and fertility. As such, *OfMasc* and *Ofdsx* constitute potential target genes in research aimed at controlling *O. furnacalis* and other lepidopteran pests (Figure S4).

The key masculinization factor played an important role and appears to have a conserved function in lepidopteran insects [21,22,23,24,30,36,43,44]. In *B. mori*, the *Masc* gene has two CCCH zinc finger domains and is regulated by *Fem* piRNA in order to control the male-specific *dsx* isoform expression, which is consistent with our results (Figures S1 and S3) [21,22,24]. In *A. ipsilon* and *P. xylostella*, the *Masc* gene was identified and shown to control masculinization through regulating the expression of *dsx* [36,44]. In previous studies, the *OfMasc* gene was cloned and shown to be regulated by *Wolbachia* to induce female-specific strains [30,31]. In our research, we used the CRISPR/Cas9 genome editing system to knock out the *OfMasc* gene, and then demonstrated that the *OfMasc* gene controls sexual dimorphism by regulating the expression of the *dsx* gene in *O. furnacalis* (Figure 1 and Figure 4).

Insect *dsx* genes are the downstream genes of the sex determination pathway; these genes are very conservative, and exhibit sex-specific splicing to generate male- (dsxM) and female-specific (dsxF) isoforms that control separate but corresponding sex-specific dimorphic traits [4,9,15,29,45]. In our study, we used the CRISPR/Cas9 genome editing system to disrupt the *Ofdsx* common region, which induced the inversion of sexual dimorphism in areas including pigmentation, the external genitals, and sex-biased genes (Figure 1, Figure 2, Figure 3, Figure 4, Figure 5 and Figure 6). In *B. mori*, *dsx* is an important transcription factor that regulates sexually dimorphic differentiation. Mutation of *Bmdsx* induced abnormal external genitals and led to female and male sterility; as such, it could act as a targeted gene for sterile insect technologies (SIT) [24,25,46]. In other lepidopteran pests, including *O. scapulalis* [47], *A. ipsilon* [48], *P. xylostella* [41] and *Hyphantria cunea* [42], *dsx* gene function is conservative and regulates sexual dimorphism. These previous reports have shown that, in lepidopteran insects, the *dsx* gene has high homology and controls sexual dimorphism through the sex determination pathway.

*Dsx* is a mimicry supergene [49,50]. In *O. furnacalis*, wing pigmentation shows sexual dimorphism: pigmentation is deeper in males than it is in females. In our study, we knocked out the *OfMasc* and *Ofdsx* genes, which caused abnormalities in the sexually dimorphic traits; moreover, there was a reversal in sex-specific wing pigmentation patterns. qRT-PCR showed some pigmentation genes were down-regulated in male mutants and up-regulated in female mutants (Figure 4 and Figure 6). These results demonstrate that the *dsx* gene can affect the expression of the genes that control sexual dimorphism in wing pigmentation patterns. In butterflies, a previous study found that females showed female-limited Batesian mimicry and displayed wing pattern polymorphism [51]. In *Papilio polytes*, this polymorphism is controlled by a single autosomal locus, dominant locus H, which consists of a series of genes that affect color patterns [52]. Moreover, a recent study of *P. polytes* has shown that the mimetic phenotype is controlled by the *dsx* gene [49,50]. SiRNA-mediated down-regulation of the *dsx* gene induced the severe repression of red spots and white pigmentation in female wing patterns [50]. In *O. scapulalis*, *Wolbachia*-infected females showed sexual mosaics, which were composed of male (darker) and female (lighter) sectors; both male and female *dsx* isoforms were also expressed in these individuals [47]. These results demonstrate that the *Osdsx* gene can control the sexual dimorphism of wing pigmentation patterns in *O. scapulalis*. 

In summary, we used the CRISPR/Cas9 genome editing system to disrupt the functions of the sex determination genes *Masc* and *dsx*. The results of our study demonstrate that *OfMasc* can regulate the expression of male *dsx* isoforms and induce male sexual phenotypes. *Dsx* performs essential functions in sexual dimorphism, and is involved in determining the morphology of external genitals and wing pigmentation patterns in *O. furnacalis*. The disruption of *Masc* induced a sex ratio imbalance and male sterility. Knocking out the *dsx* common region induced female and male sterility. These results demonstrate that *Masc* and *dsx* are potential target genes for efforts to control *O. furnacalis* and some other lepidopteran pests.

## 5. Conclusions

In this study, we investigated the function of *OfMasc* and *Ofdsx* in the lepidopteran agricultural pest *O. furnacalis*. We used the CRISPR/Cas9 genome editing system to successfully knock out *OfMasc* and *Ofdsx*. Mutation of *OfMasc* induced defects in the male external genitals, a sex ratio imbalance and male sterility. Disruptions of the *Ofdsx* common region caused sex-specific defects in the external genitals and adult sterility. In addition, we found that *OfMasc* and *Ofdsx* can regulate pigmentation genes to control wing pigmentation patterns. These results demonstrate that *OfMasc* and *Ofdsx* play key roles in sex determination and in the regulation of sexually dimorphic trails in *O. furnacalis*, and have the potential to be used in the genetic control of pests such as *O. furnacalis*.

## Figures and Tables

**Table 1:** The components of the artificial diet given to the laboratory strain of *Ostrinia furnacalis*.

Components	Weight (g) or Volume (mL)
Wheat germ powder	150
Yeast extract powder	40
Agar strip	14
Sucrose	5
Vitamin C	4
Sorbic acid	4
Methyl p-hydroxybenzoate	4
Linoleic acid	0.5
purified water	900

**Table 2:** Primers used in PCR amplification and mutant detection.

Primer Name	Primer Sequence (5′-3′)	Primer Purpose
Masc-sgF1	TAATACGACTCACTATAGGGTTTGTAGGGATTACGTTGTTTTAGAGCTAGAAATAGCAA	Preparation of sgRNA templates
Masc-sgF2	TAATACGACTCACTATAGGCGACATGAAGCTCACGGAGTTTTAGAGCTAGAAATAGCAA	
Dsx-sgF1	TAATACGACTCACTATAGGTGTCCCTAGAAACCCTGGGTTTTAGAGCTAGAAATAGCAAGTTAAAATAAG	
sgRNA-R	AAAAGCACCGACTCGGTGCCACTTTTTCAAGTTGATAACGGACTAGCCTTATTTTAACTTGCTATTTCTAGCTCTAAAAC	
Masc-F1	ACATAGTGAACAAAATGGCCGCCAC	Identification of somatic mutations
Masc-R1	TTGAGGTGGTGGTGCTGAAACAGAA	
Dsx-DC-F1	AAACGCTTTTATTTAGAGGTTAAGAGGG	
Dsx-DC-R1	GCTGAAATGATGATGATGATCCAAA	
Dsx-RTPCR-F	AAGTTCCACTATTCCTGGGAG	qRT-PCR for genes
Dsx-RTPCR-R	AGCACATCGAGTACGAGGAG	
Actin-qF	CCGTCCTCCTGACCGAGGCTC	
Actin-qR	GGTGTGGGAGACACCATCTCCG	
Vg-qF	TCTTACAAATCGCGCAATGG	
Vg-qR	GACTTGGAGACGTTCTTGAC	
OR53-qF	GGAGCTATTACCTACGTGAAGC	
OR53-qR	TTAAGCGCAGGCTGCGTTCATG	
PBP2-qF	ATGTGCTCGATGAGCGTTGT	
PBP2-qR	CTTGGATGAAAGGCAGAGGAT	
PBP3-qF	AAGACGCTTGTGGTGATGGCA	
PBP3-qR	GATCAGTTGTAATCCTGTGGC	
Optix-qF	GCCCATTATCAGGAAGCAGA	
Optix-qR	CAGCTCCCTCTTCTTTGTCG	
206617-qF	ATGGATACGAGGCACAAAGC	
206617-qR	GAGGATCAGTGTGCAAAGCA	
APA-qF	TATGGCGGTACGACACTTTG	
APA-qR	GGAAGGCAGTCCGTCTTGTA	
Ddc-qF	TTGGTTCGTCTTGAGGCTTT	
Ddc-qR	CCATTAATGCGCTTCAACAA	
Tan-qF	TCATCGCGACGTATGCTAAC	
Tan-qR	ATGGTTCCAATGAGGTCGTC	
Ebony-qF	CGTCTGCCCTATTCAGCAAT	
Ebony-qR	CACCAGCTTCTGAGGGTCTC	
Yellow-qF	TGTTGGAATTCCGCTCTTTC	
Yellow-qR	ACGGGACCGTGTAAATTCTG	

## Data Availability

All the data and resources generated for this study are included in the article and the Supplemental Materials.

## Supplementary Materials

The following supporting information can be downloaded at: https://www.mdpi.com/article/10.3390/cells11142161/s1, Figure S1: Phylogenetic analysis of the *OfMasc* gene. Figure S2: Phylogenetic relationship of insect *dsx* genes, generated using NCBI BLAST program. Figure S3: The splicing patterns of *Ofdsx* were examined by RT-PCR in wild-type and mutant insects. Figure S4: The sex ratio difference in *OfMasc* mutants and the fertility of *OfMasc* and *Ofdsx* mutants. Table S1: Mutagenesis of *OfMasc* and *Ofdsx* induced by Cas9/sgRNA.

## References

[1] Hopkins B.R., Kopp A. (2021). Evolution of sexual development and sexual dimorphism in insects. Curr. Opin. Genet. Dev..

[2] Parker G.A. (2014). The sexual cascade and the rise of pre-ejaculatory (Darwinian) sexual selection, sex roles, and sexual conflict. Cold Spring Harb. Perspect. Biol..

[3] Mank J.E. (2017). The transcriptional architecture of phenotypic dimorphism. Nat. Ecol. Evol..

[4] Salz H.K. (2011). Sex determination in insects: A binary decision based on alternative splicing. Curr. Opin. Genet. Dev..

[5] Gempe T., Beye M. (2011). Function and evolution of sex determination mechanisms, genes and pathways in insects. Bioessays.

[6] Bachtrog D., Mank J.E., Peichel C.L., Kirkpatrick M., Otto S.P., Ashman T.L., Hahn M.W., Kitano J., Mayrose I., Ming R. (2014). Sex determination: Why so many ways of doing it?. PLoS Biol..

[7] Prakash A., Monteiro A. (2016). Molecular mechanisms of secondary sexual trait development in insects. Curr. Opin. Insect Sci..

[8] Coschigano K.T., Wensink P.C. (1993). Sex-specific transcriptional regulation by the male and female doublesex proteins of Drosophila. Genes Dev..

[9] Raymond C.S., Shamu C.E., Shen M.M., Seifert K.J., Hirsch B., Hodgkin J., Zarkower D. (1998). Evidence for evolutionary conservation of sex-determining genes. Nature.

[10] Raymond C.S., Murphy M.W., O’Sullivan M.G., Bardwell V.J., Zarkower D. (2000). Dmrt1, a gene related to worm and fly sexual regulators, is required for mammalian testis differentiation. Genes Dev..

[11] Kaiser V.B., Bachtrog D. (2010). Evolution of sex chromosomes in insects. Annu. Rev. Genet..

[12] Murray S.M., Yang S.Y., Van Doren M. (2010). Germ cell sex determination: A collaboration between soma and germline. Curr. Opin. Cell Biol..

[13] Burtis K.C., Baker B.S. (1989). *Drosophila doublesex* gene controls somatic sexual differentiation by producing alternatively spliced mRNAs encoding related sex-specific polypeptides. Cell.

[14] Matson C.K., Zarkower D. (2012). Sex and the singular DM domain: Insights into sexual regulation, evolution and plasticity. Nat. Rev. Genet..

[15] Williams T.M., Selegue J.E., Werner T., Gompel N., Kopp A., Carroll S.B. (2008). The regulation and evolution of a genetic switch controlling sexually dimorphic traits in *Drosophila*. Cell.

[16] Futahashi R. (2016). Color vision and color formation in *dragonflies*. Curr. Opin. Insect Sci..

[17] Tanaka S., Harano K.I., Nishide Y., Sugahara R. (2016). The mechanism controlling phenotypic plasticity of body color in the desert locust: Some recent progress. Curr. Opin. Insect Sci..

[18] Traut W., Sahara K., Marec F. (2007). Sex chromosomes and sex determination in Lepidoptera. Sex. Dev..

[19] Fujii T., Shimada T. (2007). Sex determination in the silkworm, *Bombyx mori*: A female determinant on the W chromosome and the sex-determining gene cascade. Semin. Cell Dev. Biol..

[20] Nagaraju J., Gopinath G., Sharma V., Shukla J.N. (2014). Lepidopteran sex determination: A cascade of surprises. Sex. Dev..

[21] Kiuchi T., Koga H., Kawamoto M., Shoji K., Sakai H., Arai Y., Ishihara G., Kawaoka S., Sugano S., Shimada T. (2014). A single female-specific piRNA is the primary determiner of sex in the silkworm. Nature.

[22] Katsuma S., Sugano Y., Kiuchi T., Shimada T. (2015). Two conserved cysteine residues are required for the masculinizing activity of the silkworm Masc protein. J. Biol. Chem..

[23] Kiuchi T., Sugano Y., Shimada T., Katsuma S. (2019). Two CCCH-type zinc finger domains in the Masc protein are dispensable for masculinization and dosage compensation in *Bombyx mori*. Insect Biochem. Mol. Biol..

[24] Xu J., Chen S., Zeng B., James A.A., Tan A., Huang Y. (2017). *Bombyx mori P-element Somatic Inhibitor* (*BmPSI*) is a key auxiliary factor for silkworm male sex determination. PLoS Genet..

[25] Xu J., Wang Y., Li Z., Ling L., Zeng B., James A.A., Tan A., Huang Y. (2014). Transcription activator-like effector nuclease (TALEN)-mediated female-specific sterility in the silkworm, *Bombyx mori*. Insect Mol. Biol..

[26] Liu D., Yan S., Huang Y., Tan A., Stanley D.W., Song Q. (2012). Genetic transformation mediated by piggyBac in the Asian corn borer, *Ostrinia furnacalis* (Lepidoptera: Crambidae). Arch. Insect Biochem. Physiol..

[27] Liu Q., Hallerman E., Peng Y., Li Y. (2016). Development of Bt rice and Bt maize in China and their efficacy in target pest control. Int. J. Mol. Sci..

[28] Kageyama D., Nishimura G., Hoshizaki S., Ishikawa Y. (2002). Feminizing *Wolbachia* in an insect, *Ostrinia furnacalis* (Lepidoptera: Crambidae). Heredity.

[29] Wang X.Y., Zheng Z.Z., Song H.S., Xu Y.Z. (2014). Conserved RNA *cis*-elements regulate alternative splicing of *Lepidopteran doublesex*. Insect Biochem. Mol. Biol..

[30] Fukui T., Kawamoto M., Shoji K., Kiuchi T., Sugano S., Shimada T., Suzuki Y., Katsuma S. (2015). The endosymbiotic bacterium *Wolbachia* selectively kills male hosts by targeting the *Masculinizing* gene. PLoS Pathog..

[31] Fukui T., Kiuchi T., Shoji K., Kawamoto M., Shimada T., Katsuma S. (2018). In vivo masculinizing function of the *Ostrinia furnacalis Masculinizer* gene. Biochem. Biophys. Res. Commun..

[32] Yang B., Ozaki K., Ishikawa Y., Matsuo T. (2015). Identification of candidate odorant receptors in Asian corn borer *Ostrinia furnacalis*. PLoS ONE.

[33] Larkin M.A., Blackshields G., Brown N.P., Chenna R., McGettigan P.A., McWilliam H., Valentin F., Wallace I.M., Wilm A., Lopez R. (2007). Clustal W and Clustal X version 2.0. Bioinformatics.

[34] Tamura K., Peterson D., Peterson N., Stecher G., Nei M., Kumar S. (2011). MEGA5: Molecular evolutionary genetics analysis using maximum likelihood, evolutionary distance, and maximum parsimony methods. Mol. Biol. Evol..

[35] Wang Y., Li Z., Xu J., Zeng B., Ling L., You L., Chen Y., Huang Y., Tan A. (2013). The CRISPR/Cas system mediates efficient genome engineering in *Bombyx mori*. Cell Res..

[36] Harvey-Samuel T., Norman V.C., Carter R., Lovett E., Alphey L. (2019). Identification and characterisation of a *Masculinizer* homolog in the diamondback moth *Plutella xylostella*. Insect Mol. Biol..

[37] You L., Bi H.L., Wang Y.H., Li X.W., Chen X.E., Li Z.Q. (2019). CRISPR/Cas9-based mutation reveals *Argonaute 1* is essential for pigmentation in *Ostrinia furnacalis*. Insect Sci..

[38] Kondo S., Ueda R. (2013). Highly improved gene targeting by germline-specific Cas9 expression in *Drosophila*. Genetics.

[39] Bi H.L., Xu J., Tan A.J., Huang Y.P. (2016). CRISPR/Cas9-mediated targeted gene mutagenesis in *Spodoptera litura*. Insect Sci..

[40] Xu J., Zhan S., Chen S., Zeng B., Li Z., James A.A., Tan A., Huang Y. (2017). Sexually dimorphic traits in the silkworm, *Bombyx mori*, are regulated by *doublesex*. Insect Biochem. Mol. Biol..

[41] Wang Y., Chen X., Liu Z., Xu J., Li X., Bi H., Andongma A.A., Niu C., Huang Y. (2019). Mutation of *doublesex* induces sex-specific sterility of the diamondback moth *Plutella xylostella*. Insect Biochem. Mol. Biol..

[42] Li X., Liu Q., Liu H., Bi H., Wang Y., Chen X., Wu N., Xu J., Zhang Z., Huang Y. (2019). Mutation of *doublesex* in *Hyphantria cunea* results in sex-specific sterility. Pest Manag. Sci..

[43] Lee J., Kiuchi T., Kawamoto M., Shimada T., Katsuma S. (2015). Identification and functional analysis of a *Masculinizer* orthologue in *Trilocha varians* (Lepidoptera: Bombycidae). Insect Mol. Biol..

[44] Wang Y.H., Chen X.E., Yang Y., Xu J., Fang G.Q., Niu C.Y., Huang Y.P., Zhan S. (2019). The *Masc* gene product controls masculinization in the black cutworm, *Agrotis ipsilon*. Insect Sci..

[45] Williams T.M., Carroll S.B. (2009). Genetic and molecular insights into the development and evolution of sexual dimorphism. Nat. Rev. Genet..

[46] Tan A., Fu G., Jin L., Guo Q., Li Z., Niu B., Meng Z., Morrison N.I., Alphey L., Huang Y. (2013). Transgene-based, female-specific lethality system for genetic sexing of the silkworm, *Bombyx mori*. Proc. Natl. Acad. Sci. USA.

[47] Sugimoto T.N., Fujii T., Kayukawa T., Sakamoto H., Ishikawa Y. (2010). Expression of a *doublesex* homologue is altered in sexual mosaics of *Ostrinia scapulalis* moths infected with *Wolbachia*. Insect Biochem. Mol. Biol..

[48] Chen X., Cao Y., Zhan S., Tan A., Palli S.R., Huang Y. (2019). Disruption of sex-specific *doublesex* exons results in male- and female-specific defects in the black cutworm, *Agrotis ipsilon*. Pest Manag. Sci..

[49] Kunte K., Zhang W., Tenger-Trolander A., Palmer D.H., Martin A., Reed R.D., Mullen S.P., Kronforst M.R. (2014). *doublesex* is a mimicry supergene. Nature.

[50] Nishikawa H., Iijima T., Kajitani R., Yamaguchi J., Ando T., Suzuki Y., Sugano S., Fujiyama A., Kosugi S., Hirakawa H. (2015). A genetic mechanism for female-limited Batesian mimicry in *Papilio butterfly*. Nat. Genet..

[51] Clarke C.A., Sheppard P.M. (1960). Super-genes and mimicry. Heredity.

[52] Loehlin D.W., Carroll S.B. (2014). Evolutionary biology: Sex, lies and butterflies. Nature.

