# Supplementary material for: Masculinizer and Doublesex as Key Factors Regulate Sexual Dimorphism in Ostrinia furnacalis
Source: Cells. 2022 Jul 11;11(14):2161. doi: 10.3390/cells11142161 (PMC9320909; doi:10.3390/cells11142161)
Supplement: Supplementary file 1 [file cells-11-02161-s001.zip › cells-1815904-supplementary.pdf]

A

B

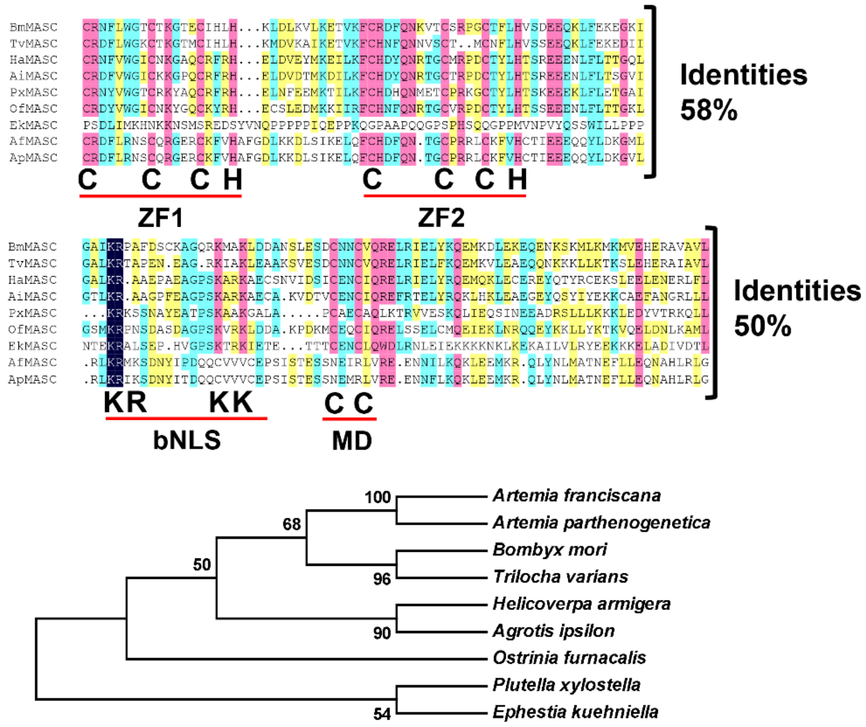

Fig.S1 Phylogenetic analysis of the *OfMasc* gene. (A) Multiple alignment of MASC protein sequences from nine species including *Bombyx mori* (BmMASC, Accession: BAO79517.1), *Trilocha varians* (TvMASC, Accession: BAS02075.1), *Helicoverpa armigera* (HaMASC, Accession: QCD63870.1), *Agrotis ipsilon* (AiMASC, Wang et al. 2019), *Plutella xylostella* (PxMASC, Harvey-Samuel et al. 2020), *Ostrinia furnacalis* (OfMASC, Accession: BAS02074.1), *Ephestia kuehniella* (EkMASC, Accession: QXE45293.1), *Artemia franciscana* (AfMASC, Accession: ARB66312.1), and *Artemia parthenogenetica* (ApMASC, Accession: ARB66313.1). The highly conserved regions are highlighted by capital letters, including CCCH-type zinc fingers (ZF), the bipartite nuclear localization signal (bNLS), and the masculinization domain (MD). (B) Phylogenetic relationships between the molecular phylogenetic analyses were conducted using the neighbor-joining method and MEGA5.1. Bootstrap values were obtained over 1000 replications.

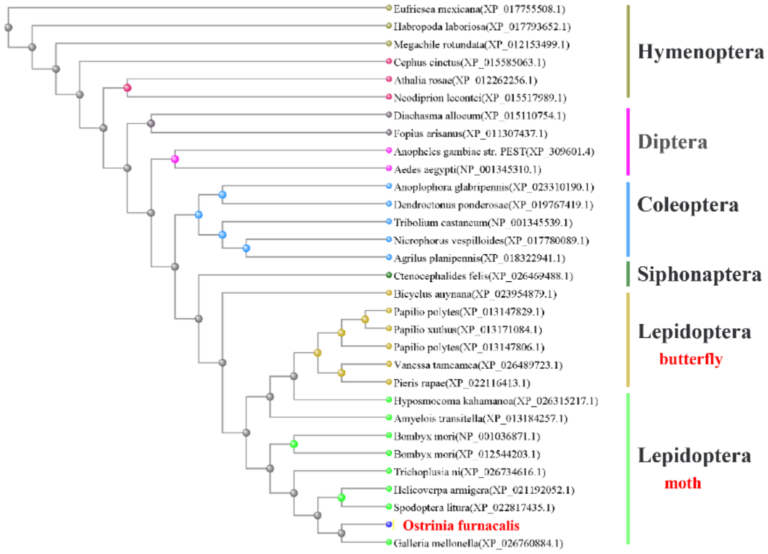

Fig.S2 Phylogenetic relationship of insect *dsx* genes, generated using NCBI BLAST program.

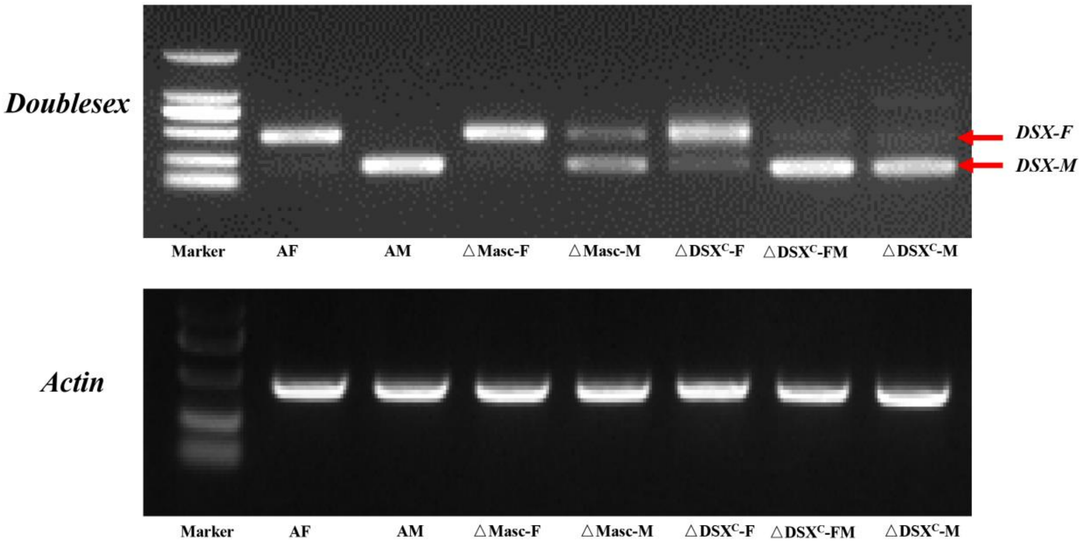

Fig.S3 The splicing patterns of *Ofdsx* were examined by RT-PCR in wild-type and mutant insects. The red arrows indicate male- and female-type splicing of *Ofdsx*. The *O. furnacalis* actin gene was used as an internal control. Adult female (AF); adult male (AM).

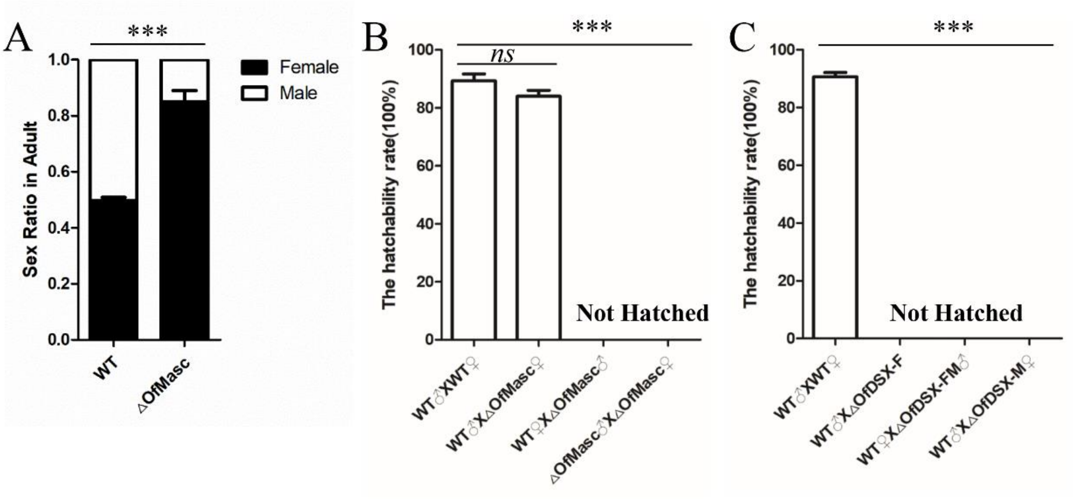

Fig.S4 The sex ratio difference in *OfMasc* mutants, and the fertility of *OfMasc* and *Ofdsx* mutants. (A) Disruption of the *OfMasc* gene induced an imbalance in the sex ratio. All data represent the average of three independent experiments ± SEM. (B) The hatchability rate when *OfMasc* mutants mated with each other. n.s., not significant; The asterisks (** or ***) indicate significant differences ($P < 0.01$ or $P < 0.001$) between adult mutants and the wild-type individuals; these were assessed using a two-tailed *t*-test. (C) The hatchability rate when *Ofdsx* mutants mated with each other. The asterisks (** or ***) indicate the significant differences ($P < 0.01$ or $P < 0.001$) between adult mutants and wild-type individuals; these were assessed using a two-tailed *t*-test.

	sgRNA/Cas9 concentration (ng/μL)	Injected eggs	Hatched larvae (%)	Live pupae (F/M)	Live adults (F/M)	Mutation phenotype of adults (F/M/FM)
OfMasc	150/300	355	121(34%)	76(65/11)	58(50/8)	6(0/6/0)
Ofdsx	150/300	468	215(46%)	123(58/65)	79(32/37)	28(9/12/7)
ddH2O	-	143	101(71%)	66(30/36)	48(23/25)	-

Table S1 Mutagenesis of *OfMasc* and *Ofdsx* induced by Cas9/sgRNA
